# High quality RNA from multiple brain regions simultaneously acquired by laser capture microdissection

**DOI:** 10.1186/1471-2199-10-69

**Published:** 2009-07-06

**Authors:** Wei-Zhi Wang, Franziska M Oeschger, Sheena Lee, Zoltán Molnár

**Affiliations:** 1 Department of Physiology, Anatomy and Genetics, University of Oxford, Le Gros Clark Building, South Parks Road, Oxford, OX1 3QX, UK

## Abstract

**Background:**

Laser capture microdissection enables the isolation of single cells or small cell groups from histological sections under direct microscopic observation. Combined with quantitative PCR or microarray, it is a very powerful approach for studying gene expression profiles in discrete cell populations. The major challenge for such studies is to obtain good quality RNA from small amounts of starting material.

**Results:**

We have developed a simple, flexible, and low-cost method for simultaneously producing RNA from discrete cell groups in embryonic day 15 mouse brain. In particular, we have optimized the following key steps in the procedure: staining, cryosectioning, storage of sections and harvesting of microdissected cells. We obtained the best results when staining 20 μm-thick sections with 1% cresyl violet in 70% ethanol and harvesting the microdissected tissue in RNA stabilization solution. In addition, we introduced three stop-points in the protocol which makes the tedious process of laser capture microdissection more flexible, without compromising RNA quality.

**Conclusion:**

Using this optimized method, we have consistently obtained RNA of high quality from all four simultaneously microdissected cell groups. RNA integrity numbers were all above 8, and long cDNA fragments (> 1.2 kb) were successfully amplified by reverse transcription PCR from all four samples. We conclude that RNAs isolated by this method are well suited for downstream quantitative PCR or microarray studies.

## Background

There has been an increasing interest in studying and comparing the gene expression profiles of small discrete cell populations. Such studies have been greatly facilitated by the recent development of laser capture microdissection (LCM) technology [1-3]. This technique allows precise separation of both single cells and small cell groups from a variety of tissues under direct microscopic observation. LCM can be combined with tracing, reporter gene expression, or with histological staining methods. However, the limiting factor of this approach is obtaining consistently high quality RNA from such small amounts of starting material.

For most applications, LCM requires staining of the tissue prior to dissection, which exposes RNA to aqueous solutions and chemical components. In addition, LCM is a lengthy process and is performed at room temperature. These factors can cause RNA degradation and have to be carefully considered when carrying out LCM experiments.

A large number of reported studies using LCM do not provide information about RNA integrity after the microdissection. Those that do assess the RNA quality have reported RNA integrity numbers (RINs) below 8, indicating that there had been at least some degree of degradation [4,5]. RIN is considered to be the most reliable method for evaluating RNA quality. RNA degradation constitutes a major problem as impaired RNA ultimately leads to biased profiling and a loss of information, especially for rare transcripts [6].

Originally LCM was developed to analyze cancer specimens. Therefore, most protocols were optimized for the dissection of tumour cells in different organs e.g. prostate or colon [4,7,8]. LCM is increasingly employed for mRNA expression studies in physiological and pathological conditions in a wide variety of tissues. It is particularly valuable to study gene expression in the central nervous system, where it allows the isolation of specific neuronal subpopulations from the surrounding heterogenous brain tissue [5,9-14].

Gene expression profiling of specific cell populations usually requires the isolation and comparison of at least two adjacent cell groups. A rigorous assessment demands that the different cell groups are collected from the same tissue sections. Obtaining equally high quality RNA from these individual populations simultaneously is an essential prerequisite to a meaningful expression profile comparison. This poses a particular challenge as the time necessary for collecting the different samples from each section increases many-fold.

In addition to the technical challenges, many researchers using LCM also face organizational difficulties. Laser capture systems which are shared among different research groups only allow time-limited access, and separation of histology from LCM facilities further complicate the management of the experiment. LCM can also be very costly, as special membrane-covered slides have to be used.

We have addressed these issues and developed a simple, convenient and low-cost method of producing high quality RNA from small cell groups, microdissected by UV laser. This method has been specifically adapted for comparing gene expression profiles in multiple adjacent cortical layers from the same embryonic brain tissue. We report here a full protocol that considerably improves RNA integrity and allows maximal flexibility in the experimental procedure.

## Results

**Figure 1:**
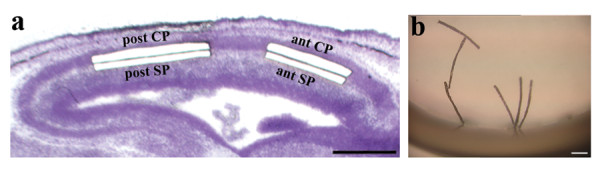
**Laser capture microdissection of four cell populations from the embryonic E15 mouse brain**. a. Subplate (SP) and lower cortical plate (CP) from anterior and posterior cortex are isolated from 20 μm cryosection using a PALM Microbeam. b. Microdissected tissue strips are harvested in RNA stabilization solution and can be visualized in the cap of the collection tube. Scalebars: a. 500 μm. b. 200 μm.

In order to optimize the protocol for gene expression analysis between multiple areas, we designed the following experimental approach (Figure 1a): anterior and posterior subplate and lower cortical plate were collected simultaneously from sections of the E15 embryonic mouse brain using a UV-laser based system. The microdissected areas had an average size of 580 μm × 40 μm. Microdissected tissue from three to four animals was pooled to increase the amount of starting material.

The following four parameters were investigated and optimized:

### 1: Staining

**Figure 2:**
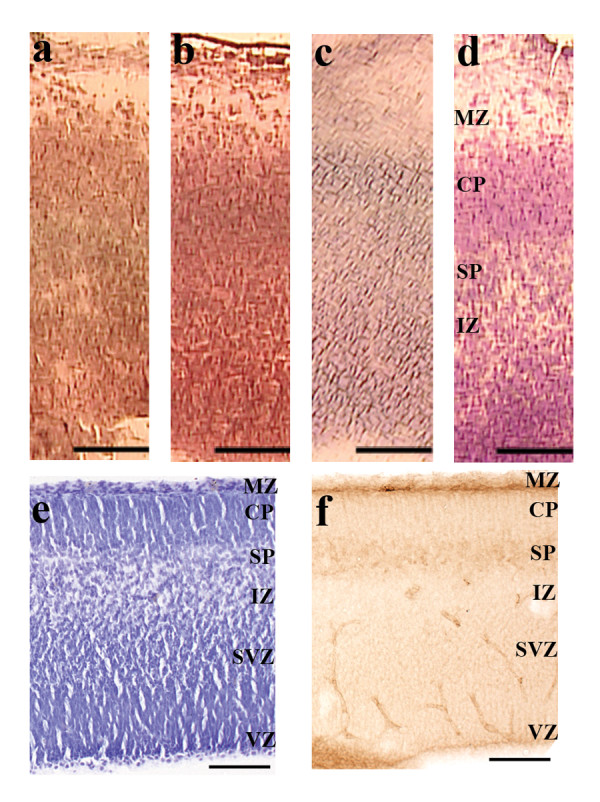
**Comparison of four staining methods**. Images were taken directly on the PALM Microbeam to demonstrate the staining and image quality available to identify the different cell populations. a. Hematoxylin. b. Hematoxylin and Eosin. c. 0.1% cresyl violet in H_2_O. d. 1% cresyl violet in H_2_O. For hematoxylin and H&E staining (a, b), sections were rinsed with 70% EtOH and H_2_O, stained with Mayer's hematoxylin for 15 sec, rinsed with H_2_O and 70% EtOH and stained with Accustain Eosin Y for 10 sec (for H&E only). For cresyl violet staining (c, d), sections were rehydrated in decreasing concentrations of ethanol, stained in cresyl violet for 45 sec and dehydrated in increasing concentrations of ethanol. 1% cresyl violet (d) provided the best morphological details and allowed clear distinction between cortical plate and subplate. e&f. Localization of the subplate at E15. Depth and width of the subplate layer on cresyl violet stained sections (e) were confirmed with immunohistochemistry against Nurr1 (f). Scalebars: 100 μm. MZ = marginal zone, CP = cortical plate, SP = subplate, IZ = intermediate zone, SVZ = subventricular zone, VZ = ventricular zone.

We compared four different staining protocols – hematoxylin, hematoxylin and eosin (H&E), 0.1% cresyl violet and 1% cresyl violet (Figure 2a–d). We found that either hematoxylin only or H&E resulted in a faint and nearly uniform staining pattern throughout the cortex, and the different cortical layers were difficult to distinguish (Figure 2a, b). Cresyl violet staining gave overall better contrast and more morphological details than the hematoxylin stains (Figure 2c, d). This staining allowed us to reliably identify the dense and darkly stained cortical plate and the more cell-sparse and lightly stained subplate just below. The location and width of the subplate layer was confirmed with immunohistochemistry against Nurr1 (Nuclear receptor related 1 protein), a known subplate marker expressed during embryonic and postnatal ages (Figure 2e, f) [15,16]. To achieve satisfactory staining results, 1% cresyl violet required a much shorter incubation time (~30 secs) than 0.1% cresyl violet (~2 min). In order to prevent possible RNA degradation during a longer staining period, 1% cresyl violet was used in our optimized protocol.

As it had been previously reported that ethanol-based staining solutions improve RNA integrity [4], we also prepared 1% cresyl violet in 70% EtOH. We found that this ethanolic staining solution provided equally good results as the water-based solution (data not shown). We also noted that xylene was not necessary to achieve clear tissue morphology but instead often led to subsequent darkening of the sections (data not shown).

### 2: Thickness of cryosections

**Figure 3:**
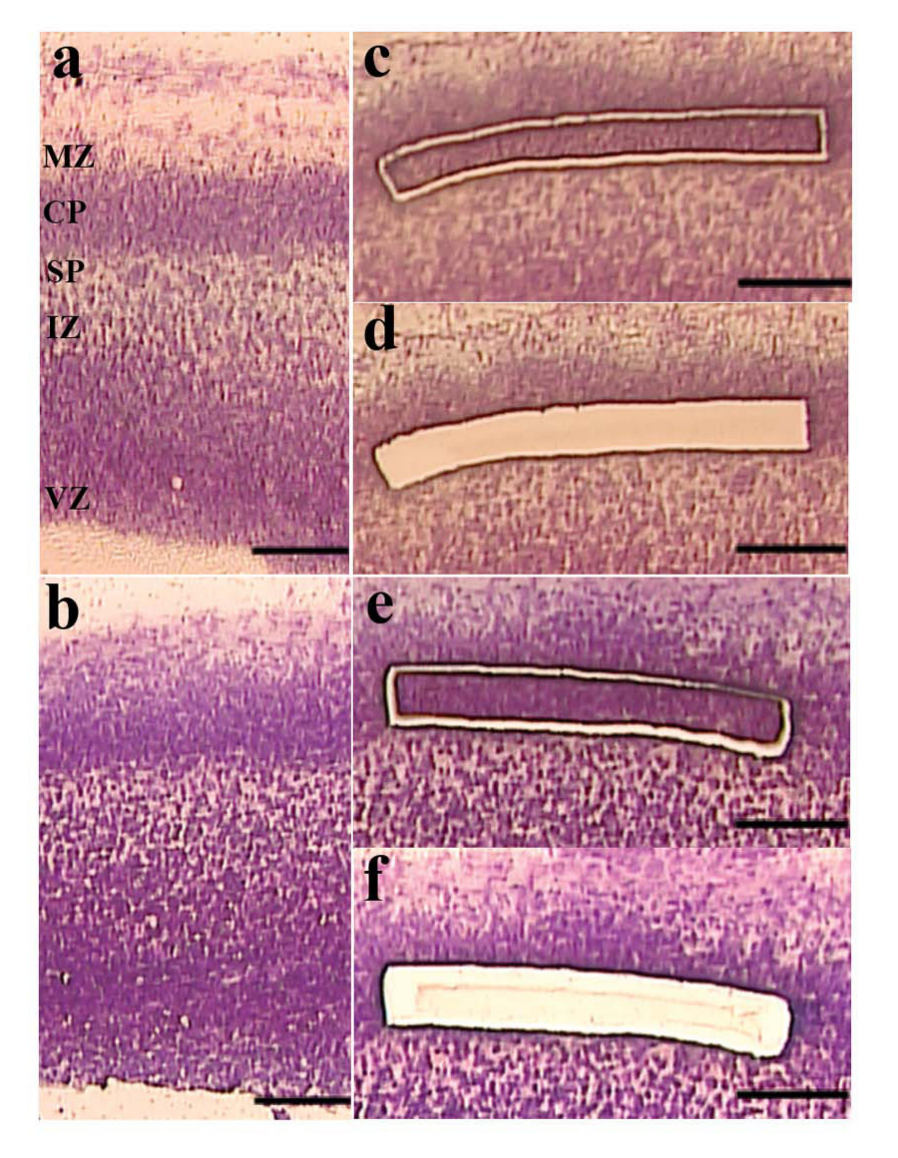
**Comparison of two cryosection thicknesses – 10 μm (a, c, d) and 20 μm (b, e, f)**. 10 μm (a) and 20 μm (b) sections showed tissue morphology of similar quality when stained with 1% cresyl violet. A slightly higher UV laser energy was needed for cutting the 20 μm sections. However, increasing thickness from 10 μm (c, d) to 20 μm (e, f) did not cause increased tissue damage during laser cutting (c, e) and catapulting (d, f). Scalebars: 100 μm. MZ = marginal zone, CP = cortical plate, SP = subplate, SVZ = subventricular zone, VZ = ventricular zone.

Manufacturer's guidelines and many protocols recommend cutting 5–12 μm sections [13,17]. We tested two different section thicknesses for LCM: 10 μm and 20 μm. Cresyl violet staining resulted in equally clear morphological details for sections with both thicknesses (Figure 3a, b). During LCM, we used slightly higher laser energy to cut the 20 μm sections than the 10 μm (UV energy of 76 points vs. 72 points, respectively), and cut around the cell group two or three times. With these minor adaptations, both cutting and catapulting were consistently successful. We did not notice any increase in burnt or damaged tissue on the 20 μm sections compared to the 10 μm sections, neither after cutting (Figure 3c, e) nor after catapulting (Figure 3d, f).

We further verified whether efficient cutting and catapulting was also possible for other tissue types on the same 20 μm sections (data not shown). We found that the laser parameters used for cortex could be applied to other nervous tissues (brain stem, cerebellum and thalamus), whereas tongue muscle and cartilage could be successfully microdissected after some minor adjustments to the laser settings (UV energy of 78 vs. 76 points).

### 3: Storage of cut sections

**Figure 4:**
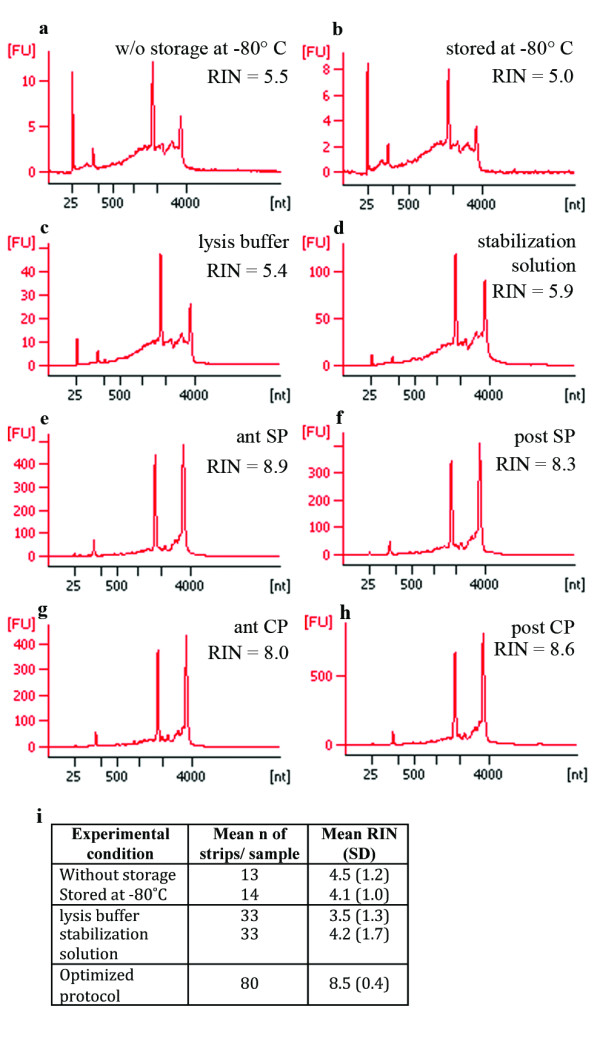
**RNA quality after different processing conditions was analysed on Agilent RNA 6000 Pico Chips**. For each approach, the RNA trace with the highest RIN out of four samples is shown here. The y-axis of the electropherograms represents fluorescence units (FU) and the x-axis represents the nucleotide length of the RNA (nt). The peaks of the 18S and 28S rRNA fragments are clearly visible. a&b After staining and dehydration, cryosections were either processed immediately by LCM (a), or they were stored at -80°C for several days before LCM (b). Storage of sections only had a minor effect on RNA integrity but greatly improved the flexibility of the workflow. c&d Microdissected cell groups were either harvested in lysis buffer (c) or RNA stabilization solution (d). Stabilization solution slightly improved the RNA integrity and in addition allowed visualization of the dissected strips. e-h. The final optimized protocol yields RNA of excellent quality with RINs > 8 from all four microdissected cell groups: anterior subplate (SP)(e), posterior SP (f), anterior cortical plate (CP)(g), posterior CP (h). i. Summary of the different experimental conditions, the average number of strips processed per area, and the average and standard deviation (SD) of the RINs among the four groups after LCM.

We tested whether storage of sections at -80°C before LCM would noticeably impair RNA quality as previously suggested [9] by comparing two approaches. For the first, two slides were prepared at a time, and cell groups were laser capture microdissected immediately after staining and dehydration. For the second approach, stained and dehydrated slides were stored in a box with desiccant at -80°C for several days. We noted that condensation accumulated on slides when they were brought from -80°C to room temperature, which might impair RNA integrity due to RNase activity in rehydrated tissue. This problem could be simply prevented by allowing the slides to warm up in a tube containing desiccant beads. We obtained RNA with similar RINs from both approaches with an average RIN of 4.5 for unstored sections and of 4.1 for stored sections (Figure 4a, b, i). As the ability to store slides allows a more manageable and flexible workflow, we incorporated this approach in our final protocol.

### 4: Collection of dissected tissue

Collecting samples from multiple regions of the same brain section and pooling samples from several slides increases the time the tissue is in the harvesting tube. We compared collection of tissue in RNA stabilization solution and lysis buffer (Figure 4c, d, i). Samples preserved in stabilization solution yielded slightly better RNA quality (average RIN = 4.2) than when dissected tissue was collected directly in lysis buffer (average RIN = 3.5).

**Figure 5:**
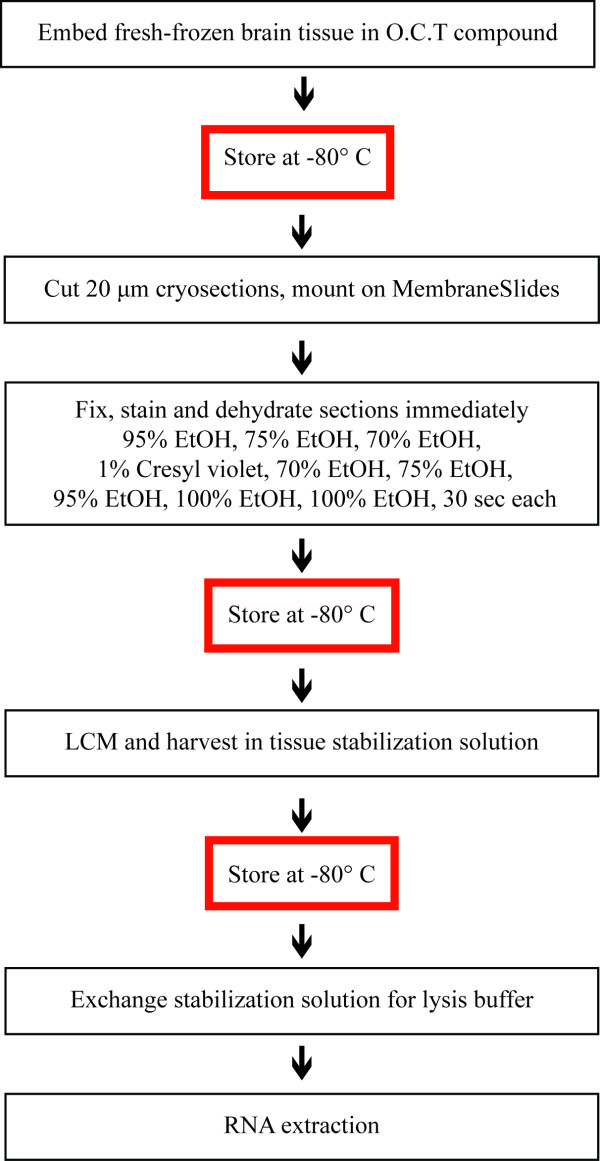
**Flow diagram of the optimized protocol with possible stop-points (red boxes)**. All steps were performed in an RNase -free environment. Fresh-frozen whole heads were stored at -80°C for up to several months. Heads were cryosectioned at 20 μm, and four to six sections were mounted on a membrane-coated PEN slide. Sections were fixed in 95% EtOH, rehydrated with 75% and 70% EtOH, stained with 1% cresyl violet in 70% EtOH and dehydrated with 70%, 75%, 95%, 100%, 100% EtOH, 30 sec each. Slides were stored at -80°C up to several weeks in a box with desiccant beads. Four cell groups from the same section were microdissected, harvested in individual tubes with RNA stabilization solution and stored at -80°C. Stabilization solution was replaced with lysis buffer and cell lysates from four brains were pooled before RNA extraction.

The complete optimized protocol is summarized in Figure 5. Using this protocol, we obtained RNA of excellent quality from all four cell groups and importantly reduced the variability between samples. Quality was assessed using the RIN, which has been reported as the most reliable method for evaluating RNA quality in LCM samples [5]. RIN is based on a software algorithm developed by Agilent Technologies that determines RNA integrity using multiple features of the electropherogram [18]. The four cell groups collected had RINs ranging from 8.0 to 8.9 (Figure 4e–i), indicating that RNA is of particularly high quality for downstream applications [6].

**Figure 6:**
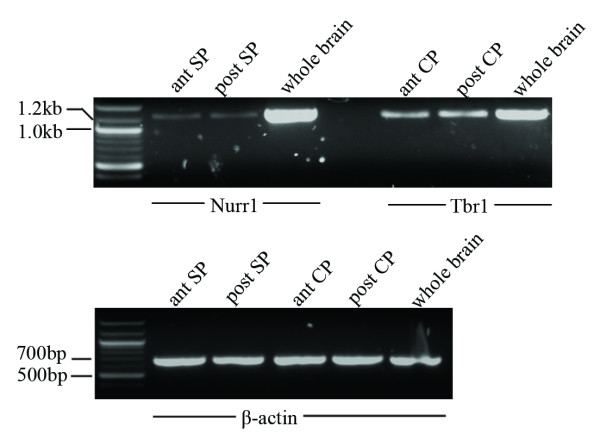
**RT-PCR analysis to confirm the presence of selected long transcripts as an additional RNA quality control**. Total RNA from the whole brain was used as a positive control. a. A 1244 bp fragment of Nurr1 amplified from anterior and posterior subplate; a 1204 bp fragment of Tbr1 amplified from anterior and posterior cortical plate. b. A 677 bp long fragment of β-actin transcript was present in all four samples.

In addition to the RIN, we amplified long fragments from selected genes (β-actin, Nurr1 and Tbr1) by RT-PCR analysis (Figure 6). The presence of long transcripts is an additional indicator of the RNA integrity. The housekeeping gene β-actin was used as a control for the general integrity of the RNA and efficiency of the RT reaction. From all four samples, a 677 bp long β-actin PCR product was generated (Figure 6b). Nurr1 is specifically present in the subplate at E15 (Figure 2f) and later stages [15,16] while Tbr1 (T-box brain gene 1) is strongly expressed in the deep layers of the cerebral cortex throughout development [19]. A 1244 bp long cDNA fragment of Nurr1 and a 1204 bp long fragment of Tbr1 were amplified from the subplate and the cortical plate samples, respectively (Figure 6a). This indicates that these long transcripts were successfully isolated from all four laser microdissected samples and that RNA degradation was minimal.

RNA yields from LCM samples are difficult to evaluate as concentrations are usually below the detection limit of spectrophotometers (10 ng/μl), and bioanalyzers only give an inaccurate estimate of quantity at this concentration. In order to estimate RNA quantities more reliably, we microdissected and pooled 20 μm-thick strips from approximately 140 sections. RNA extracted from this large number of tissue samples was successfully quantified by spectrophotometry, and we obtained yields of total RNA between 120 ng (subplate) and 180 ng (cortical plate) per sample. Performing cell counts on cresyl violet stained material, we estimated that each microdissected strip (580 μm × 40 μm) contains approximately 600 cells in the cortical plate and 300 cells in the subplate at E15. This results in an average RNA yield of 2.5 pg per cell.

## Discussion

One of the major difficulties when using LCM for gene expression profiling is to obtain RNA of good quality for subsequent microarray analysis. This is particularly challenging if multiple cell groups are dissected simultaneously from the same tissue sections as in this study. In addition, LCM can be very time-consuming and difficult to manage, especially if complex logistics are involved (i.e. scheduling shared laser capture microdissector, transport between facilities). Finally, LCM may be expensive if a large number of membrane-coated slides or special commercial kits are used. LCM requires a combination of critical procedures including tissue collection, cryosectioning, fixation/staining/dehydration, storage and microdissection. Each can have a serious impact on the subsequent RNA quality. To address these issues, we have analyzed and optimized four crucial steps in the LCM procedure: tissue staining and dehydration, section thickness, storage of sections and harvesting and storage of microdissected samples.

The ability to accurately identify different cell groups is essential in most LCM studies. Optimal staining allows clear visualization of the histological structure and at the same time minimizes RNA degradation. For the latter, all staining protocols have to be very short to reduce the time the tissue is exposed to room temperature, aqueous solutions and chemical components. We evaluated four different staining methods – hematoxylin, H&E, 0.1% cresyl violet and 1% cresyl violet. Hematoxylin and H&E were tested because RNAse inhibitors can be added to the solutions which would potentially reduce RNA degradation [7]. We found that staining with 1% cresyl violet needed the shortest staining time (30 seconds) while at the same time provided the best histological details (Figure 2d). As RNAse inhibitors were reported to be incompatible with cresyl violet [6], we prepared 1% cresyl violet in 70% EtOH to reduce aqueous exposure and subsequent RNA degradation.

In most studies, LCM is performed on very thin sections (5–12 μm) [13,17]. By increasing the tissue thickness, the amount of starting material can be augmented without increasing the number of slides. We tested whether it was possible to increase the section thickness without impairing staining quality and cutting and catapulting efficiency. We found that the 20 μm sections allowed equally clear visibility of the different cortical layers as 10 μm sections when stained with cresyl violet (Figure 3a, b). In addition, efficient, non-damaging cutting and catapulting with the LCM was achieved on the 20 μm sections after a few minor adjustments (Figure 3e, f). Different types of embryonic tissue including brain, tongue muscle and cartilage were successfully microdissected. The time necessary for laser-cutting increased as we had to cut around each strip at least twice. However, this did not considerably increase the overall time per slide as cutting is very rapid, nor did it result in a noticeably decrease of RNA integrity. Doubling the section thickness allowed us to half the number of sections and thus the number of slides, which is both time- and cost-effective.

It is important to note, however, that increased tissue thickness requires a lower numerical aperture and results in a broader, less precise laser beam. This effect is not noticeable when collecting relatively large areas as in our study but has to be considered when aiming to microdissect very small cell groups or single cells.

Previous studies have reported that cryosections should be processed by LCM immediately after staining to avoid RNA degradation [9]. This approach may be ideal, but it is difficult to manage, especially if LCM and histology facilities are not in the same vicinity. We found that storage of sections had only a minor effect on RNA integrity. We therefore subsequently stored sections at -80°C up to several weeks which allowed a much more flexible workflow.

It took us 30–40 minutes to harvest four cell groups from the 4–6 sections on each slide. For tissue collection, one tube was placed on the laser microdissector at room temperature while the three tubes for the other three cell groups were kept on ice. In each LCM session, six to eight slides were processed, and samples were collected in the same four tubes. Under these circumstances, we found that RNA stabilization solution preserved RNA better and yielded slightly better RNA quality than when dissected tissue was collected directly in lysis buffer. In addition, the stabilization solution allowed us to visualize and to confirm the number of dissected strips after tissue harvesting and before RNA extraction (Figure 1b). We estimate that approximately 10% of microdissected tissue was not successfully captured in the harvesting cap.

Although recently developed amplification methods make it possible to analyze degraded samples, we believe that part of the gene expression information will be lost, especially from rare transcripts [6]. In addition, RNA quality has to be consistent between different cell populations in order to allow unbiased comparison. By using our optimized protocol, we have consistently obtained RNAs with RINs > 8 from all dissected cell groups. In addition, long transcripts (> 1.2 kb) of layer-specific genes were amplified from all four samples by RT-PCR. These findings confirm that RNA quality obtained by this protocol is well suited for subsequent microarray analysis.

Currently, there is no reliable method for measuring low concentrations of RNA (< 10 ng/μl). In order to estimate the RNA yield, we microdissected a large number of strips and quantified the extracted RNA by spectrophotometry. We found an approximate yield of 1.3 ng of total RNA per cortical plate strip and 0.85 ng per subplate strip, which is consistent with the different cell densities of these two layers. Based on cell counts using cresyl-violet stained sections, we estimated an RNA recovery of 2.5 pg per cell. This is within the range of yields previously reported for brain cells collected by LCM [20,21]. It is evident, however, that the amount of RNA recovered per cell not only depends on the isolation method but also on the cell size, type and age, and therefore has to be individually determined for different tissue types.

With the recent development of very efficient amplificiation methods, it has become possible to perform microarray analysis on very small quantities of total RNA, starting from as little as 500 picograms. In order to obtain more robust results and to avoid loss of rare transcripts, we believe it is desirable to use at least 10–20 ng of RNA as starting material, if time and material limitations allow for it. Based on our estimates of RNA recovery, this corresponds to 5000 to 10,000 brain cells, or 20–30 microdissected strips in our experiment.

The optimized method described here allows a flexible workflow as not only tissue blocks but also sections and microdissected tissue can be stored at -80°C for at least several weeks. We believe that this is particularly important as many research groups only have access to shared LCM facilities which can be used sporadically. In addition, all the steps in this protocol except for the RNA extraction are performed with routine lab equipment and solutions, which makes this approach very cost-effective and easily attainable.

## Conclusion

We describe a protocol for UV-laser capture microdissection of multiple specific cell groups from the same tissue section. In particular, we have optimized four crucial procedures: 1) staining and dehydration of the tissue, 2) section thickness, 3) storage of sections, 4) harvesting and storage of microdissected cells with possible points of interruption. Using this protocol, we have consistently obtained RNA of very high quality (RINs > 8) from all simultaneously harvested cell groups and long PCR products were successfully amplified from the transcripts.

## Methods

### RNase-free experimental environment

All procedures were performed in an RNase-free environment. Working surfaces were treated with RNase decontamination solution (RNaseZap, Ambion) and rinsed with RNase-free water. Glassware was baked at 280°C for 4 h to inactivate RNase. Certified RNase-free plasticware was used in each process. All solutions were made from chemicals of molecular biology grade and RNase-free water. No additional RNase inhibitors were added into the individual solutions.

### Tissue preparation

Time-pregnant mice were killed by cervical dislocation in accordance with the UK Animals (Scientific Procedures) Act (1986). Whole heads of embryonic day 15 (E15) mice were flash-frozen directly in isopentane on dry-ice. Once hardened, the heads were embedded in O.C.T compound (Tissue-Tek) and stored at -80°C. The tissue was sectioned (10 μm or 20 μm) on a cryostat (Jung CM3000, Leica) and mounted on membrane-coated 1 mm PEN slides (Zeiss). Before use, the slides had been treated with RNase decontamination solution and UV irradiation at 320 nm for 30 min as recommended by the manufacturer. Once the first section had been mounted, the slide was kept inside the cryochamber (-18°C) while the next section was cut. Four to six sections were mounted in this way on each slide. Cutting and mounting was performed as quickly as possible to ensure that all sections adhered properly to the membrane. Two slides were prepared at a time and processed in parallel.

Sections were allowed to air-dry for at least 2 min before fixation in 95% EtOH for 30 sec, both in the cryochamber.

### Staining procedures

Hematoxylin and hematoxylin and eosin (H&E) staining were performed as previously reported for LCM samples [22]. Briefly, sections were rinsed with 70% EtOH and then with H_2_O, stained with Mayer's hematoxylin (Sigma) for 15 sec, rinsed with H_2_O followed by 70% EtOH, stained with Accustain Eosin Y (Sigma) for 10 sec (for H&E only), dehydrated with 95% EtOH, 10 sec, 100% EtOH, 40 sec, and cleared with xylene, 60 sec.

For cresyl violet staining, sections were rehydrated in 95% EtOH, 75% EtOH, 70% EtOH, stained in cresyl violet for 30–45 sec and dehydrated in 75% EtOH, 95% EtOH, 100% EtOH, 100% EtOH, 30 sec each. 0.1% cresyl violet was prepared in H_2_O and 1% cresyl violet in H_2_O or 70% EtOH, filtered and stored at 4°C for up to one month.

100% EtOH was stored with desiccant beads (Molecular sieves 4 Å beads, 8–12 mm mesh, Sigma) to prevent any rehydration.

After dehydration, sections were air-dried and either processed immediately or stored in a box with desiccant at -80°C for up to several weeks.

### Immunohistochemistry

Frozen sections were postfixed in 4% paraformaldehyde in PBS for 20 min and quenched in 1.5% hydrogen peroxide for 30 min. Sections were then blocked for 2 h at RT with 5% donkey serum (Sigma, UK) in Tris-buffered saline (TBS) with 0.1% Triton-X100 (BDH, Poole, UK) and incubated with goat anti-Nurr1 (AF2156, R&D Systems) 1/200 in 1% donkey serum in TBS at 4°C overnight. Biotinylated donkey anti-goat (Abcam) 1/500 in 1% donkey serum in TBS was applied for 2 h at RT and reacted with avidin-biotinylated enzyme complex (ABC) using the Vectastain Elite kit (Vector, UK) and diaminobenzidene (DAB) according to the manufacturer's instructions.

### Laser capture microdissection

LCM was performed using a PALM Microbeam IP 230V Z (Zeiss).

Slides which had been stored at -80°C were transported and kept in a box with desiccant on dry ice. Slides were subsequently dried one at a time in a 50 ml Falcon tube with desiccant at room temperature. From each section, all four areas were cut and catapulted into separate caps (0.6 ml Eppendorf tube). Caps either contained 50 μl of RNA stabilization solution (RNAlater, Ambion) or 50 μl lysis buffer (RLT, RNeasy micro Kit, Qiagen). Tubes were kept on ice with the cap down until all areas were collected. The approximate time required to collect all four areas from one slide (4–6 sections) was 40 min. The tissue strips that were in RNA stabilization solution were directly stored at -80°C with the cap down. Strips in lysis buffer were vortexed for 45 sec, spun down and stored at -80°C.

In order to harvest enough tissue for downstream microarray analysis, we collected the same cell groups from 3–4 brains. Sections from each brain were processed in a different LCM session and thus collected in a separate cap.

### RNA extraction, quantification and quality control

Cell groups collected in lysis buffer were defrosted on ice and pooled with the same cell lysates from different brains. Tissues stored in RNA stabilization solution were defrosted on ice and stabilization solution was replaced with lysis buffer under a dissecting microscope using a syringe. After 5 min incubation, the samples were vortexed for 45 sec and spun down. Then the same cell lysates derived from different brains were pooled.

Total RNA was extracted using the RNeasy micro kit (Qiagen) following the manufacturer's instruction. On-column DNase digestion step was performed. RNA quantities were determined using spectrophotometry (Nanodrop ND-1000, Labtech). RNA integrity after LCM was analyzed both on RNA 6000 Pico Chips (Agilent Technologies) and by reverse-transcription PCR (RT-PCR).

Approximately 20 ng of total RNA were used as a template for reverse transcription using Superscript III Reverse Transcriptase together with random hexamers (Invitrogen, Paisley, UK) following the manufacturer's instructions. Long PCR fragments corresponding to the regions of the mouse *Nurr1 *(subplate marker), *Tbr1 *(cortical plate marker) *and β-actin *cDNAs were amplified using the following sets of forward (F) and reverse (R) primers:

**Nurr1 **(1244 bp):

F 5'-CATGGACCTCACCAACACTG-3'; R 5'- CTGGGTTGGACCTGTATGCT-3'

**Tbr1 **(1204 bp):

F 5'- TCTCGACCACTGACAACCTG-3'; R 5'- GCGTAGTTGCTCACGAACTG-3'

**β-actin **(677 bp):

F 5'- AGCCATGTACGTAGCCATCC-3'; R 5'- ACATCTGCTGGAAGGTGGAC-3'PCR products were visualized on a 1.2% agarose gel. DNA molecular weight marker 1 kb (New England BioLabs, UK) was used to determine product size.

### Imaging

Images were taken on the PALM Microbeam IP 230V Z (Zeiss) or on a DMR transmission light microscope (Leica, Figure 1a and 2e, d). Brightness and contrast of images were adjusted for publishing using Adobe Photoshop CS3.

## Authors' contributions

W-ZW and FO developed and optimized the described method, performed all the experiments and wrote the manuscript. SL analyzed the RNA qualities and quantities and provided useful suggestions. ZM conceived of the study and contributed to the planning of the experiments and to the writing of the manuscript. All authors read and approved the final manuscript.
